# Supplementary material for: Content-rich biological network constructed by mining PubMed abstracts
Source: BMC Bioinformatics. 2004 Oct 8;5:147. doi: 10.1186/1471-2105-5-147 (PMC528731; doi:10.1186/1471-2105-5-147)
Supplement: Additional File 2 — The original results of the above study (non-essential files are deleted to keep the file size under the limit set by BMC bioinformatics). [file 1471-2105-5-147-S2.bz2 › chilibotAdditionalFile2/dip30/280ID2498083E1103/html/FOSB_JUNB.html]

 


 **FOSB** and **JUNB** 
  
Found 123 abstracts in PubMed, retrieved 30.  
 

 What does Google say? 
 PDF only 
| .edu only 

---

**Interactive relationship** (e.g. stimulation, inhibition, etc)

**Neutral relationship**- Serum response factor is a transcription factor, which binds to a serum response element associated with a variety of genes including immediate early genes such as c fos,  **fosB** ,  **junB** , egr 1 and 2, neuronal genes such as nurr1 and nur77 and muscle genes such as actins and myosins.  Ref: 12120892 J Physiol Pharmacol, 2002
- Nuclear extracts isolated from hypoxic rat cardiac fibroblasts contained Fra1,  **JunB**  and also included  **FosB** .  Ref: 12371906 Biochem J, 2003

**Non-interactive relationship** (e.g. studied together, co-existance, homology, etc.)

- Phosphorylated c Jun,  **JunB** , JunD, and Fra 1, but not c Fos,  **FosB** , or ATF 2, are detected in the AP 1 DNA binding complex in cells exposed to trivalent arsenicals.  Ref: 12475910 FASEB J, 2003
- The Wnt mediated repression of c Fos,  **FosB** , and  **JunB**  expression was consistent with a decrease in their binding to an AP 1 promoter element and decreased target gene transcription.  Ref: 11751871 J Biol Chem, 2002
- Forty six globes were processed for in situ hybridization with oligonucleotide probes for c fos,  **fosB** , c jun,  **junB**  and junD mRNAs, and 60 globes were immunohistochemically analysed using anti c Fos and anti c Jun antibodies.  Ref: 11825018 Exp Eye Res, 2001
- The DNA binding activities of c Fos,  **FosB**  Delta  **FosB** , Fra 1, Fra 2, and  **JunB**  were up regulated whereas JunD activity was decreased.  Ref: 11854297 J Biol Chem, 2002
- Using DNA protein gel shift assays we determined that nerve growth factor stimulates AP 1 binding in both PC12 and B5P cells, and identified c Fos,  **FosB** , Fra 1, Fra 2, c Jun,  **JunB**  and JunD in AP 1 complexes.  Ref: 11905996 J Neurochem, 2002
- Immunoblot analyses confirmed that c Jun, JunD,  **JunB** , c Fos, Fra 1, Fra 2 and  **FosB**  immunoreactive proteins were present in whole cell extracts of all antral follicles and midluteal phase corpora lutea as well as granulosa cells isolated from different sized antral follicles.  Ref: 12606371 Biol Reprod< MedlineTA>Biol Reprod, 2002
- **FosB** , Fra 2, c Jun,  **JunB**  and, most abundantly, JunD bind the AP 1 EP in the absence and presence of serotonin.  Ref: 11162889 Mol Cell Endocrinol, 2000
- Further analysis demonstrated that serum or 12 O tetradecanoylphorbol 13 acetate activation of several immediate early genes including fos,  **fosB** ,  **junB** , and egr1 was inhibited by Wnt signaling.  Ref: 11751871 J Biol Chem, 2002
- c Fos,  **FosB**  Delta  **FosB** , and  **JunB**  were associated with Smad4.  Ref: 11854297 J Biol Chem, 2002
- We found that there is a lack of induction of c Fos,  **FosB** , Fra 2 and  **JunB**  by acute cocaine exposure, and of DeltaFosB by repeated cocaine administration in both the NAc and CPu of D1 receptor mutant mice compared with wild type control mice.  Ref: 12354293 J Neurochem, 2002
- The expression of fos,  **fosB** ,  **junB** , and egr1 was also repressed in human colon tumors relative to patient matched normal tissue.  Ref: 11751871 J Biol Chem, 2002
- Expression of egr 1, egr 2, egr 3, c jun,  **junB** , c fos,  **fosB** , fra 1, fra 2, and CREB genes was analyzed by reverse transcription polymerase chain reaction in Schwann cells isolated from neonatal rat sciatic nerves and in the cell lines MSC 80, NIH 3T3, and CHO.  Ref: 11460264 Glia, 2001
- We have investigated the expression of the AP 1 transcription factor proteins, fos,  **fosB** , fra1, fra2, jun,  **junB** , junD, using Western blot analysis, in several types of asynchronously proliferating cells.  Ref: 11809534 Cancer Lett, 2002
- Expression of c fos,  **fosB** , fra 1, fra 2, and  **junB**  was low during early gestation, with a 5 to 10 fold increase on Day 23 during labor, and returned to low levels 1 day postpartum.  Ref: 12080023 Biol Reprod, 2002
- Fra 1, Fra 2,  **FosB** ,  **JunB** , JunD, c Jun, and c Fos levels are increased by EGCG treatment, as is AP1 factor binding to hINV promoter AP1 site.  Ref: 11698415 J Biol Chem, 2002
- Similarly, in the second experiment it was found that the D1R dependent induction by AMPH of Fos,  **FosB** , and  **JunB** , but not NGFI A, in the NAc was enhanced in rats exposed 1 week earlier to repeated VTA AMPH.  Ref: 12112395 Synapse, 2002
- Among the early response genes analyzed, c myc,  **junB** , junD, c jun, c fos,  **fosB** , fra, as well as max, mad1 4, sin3, only c jun and fra 2 mRNAs were up regulated after 5D exposure.  Ref: 12648525 J Steroid Biochem Mol Biol, 2003
- These studies demonstrate that a functional AP 1 site mediates MMP 2 transcription in cardiac cells through the binding of distinctive Fra1  **JunB**  and  **FosB**   **JunB**  heterodimers.  Ref: 12371906 Biochem J, 2003
- We further studied the expression of  **fosB** , c jun and  **junB** , in the AP 1 complex.  Ref: 12820376 Anticancer Res< MedlineTA>Anticancer Res, 2003
- This effect was specific because palytoxin had little effect on c Jun,  **JunB** , JunD,  **FosB** , Fra 1, or Fra 2 binding or on overall levels of transcription factor binding.  Ref: 12460732 Toxicol Appl Pharmacol, 2002
- Administration of progesterone beginning on Day 20 prevented the onset of labor and blocked the expected rise in c fos,  **fosB** , fra 1, fra 2, and  **junB**  expression on Day 23.  Ref: 12080023 Biol Reprod, 2002
- Supershift experiments and Western blots confirmed that the binding of c Fos,  **JunB** , and JunD, but not of  **FosB** , was reduced in anergized cells.  Ref: 11685114 Transplantation, 2001
- MafG, ATF2,  **FosB** , and  **JunB**  were also detected in the arsenite complex.  Ref: 12220541 Arch Biochem Biophys, 2002
- The defect is secondary to an inability to phosphorylate CREB and to induce CREB dependent immediate early genes, including c jun,  **fosB** , fra2, and  **junB** , which are required for cytokine gene induction.  Ref: 11739719 Mol Cell Biol, 2002
- Normal lens epithelial cells expressed mRNA signals for junD, but not for c fos,  **fosB** , c jun, and  **junB** . mRNAs for c fos,  **fosB** , c jun, and  **junB**  were detected in the whole lens epithelium from the vicinity to the wound to the equator from 30 min to 8 hr post injury with their peaks after 30 min to 1 hr, but were no longer detected at 10 hr or later.  Ref: 11825018 Exp Eye Res, 2001
- Co transfection of cardiac fibroblasts with Fra1  **JunB**  and  **FosB**   **JunB**  expression plasmids led to significant increases in transcriptional activity.  Ref: 12371906 Biochem J, 2003
- In unilaterally pregnant rats, stretch imposed by the growing fetus was found to increase the expression of c fos,  **fosB** , fra 1, fra 2, and  **junB**  only in the gravid horn on the day of labor.  Ref: 12080023 Biol Reprod, 2002
